# Supplementary figures and images for: Src-dependent tyrosine-phosphorylation of NM2A has a protective role against bacterial pore-forming toxins
Source: PLoS Pathog. 2026 Feb 23;22(2):e1013945. doi: 10.1371/journal.ppat.1013945 (PMC12959841; doi:10.1371/journal.ppat.1013945)

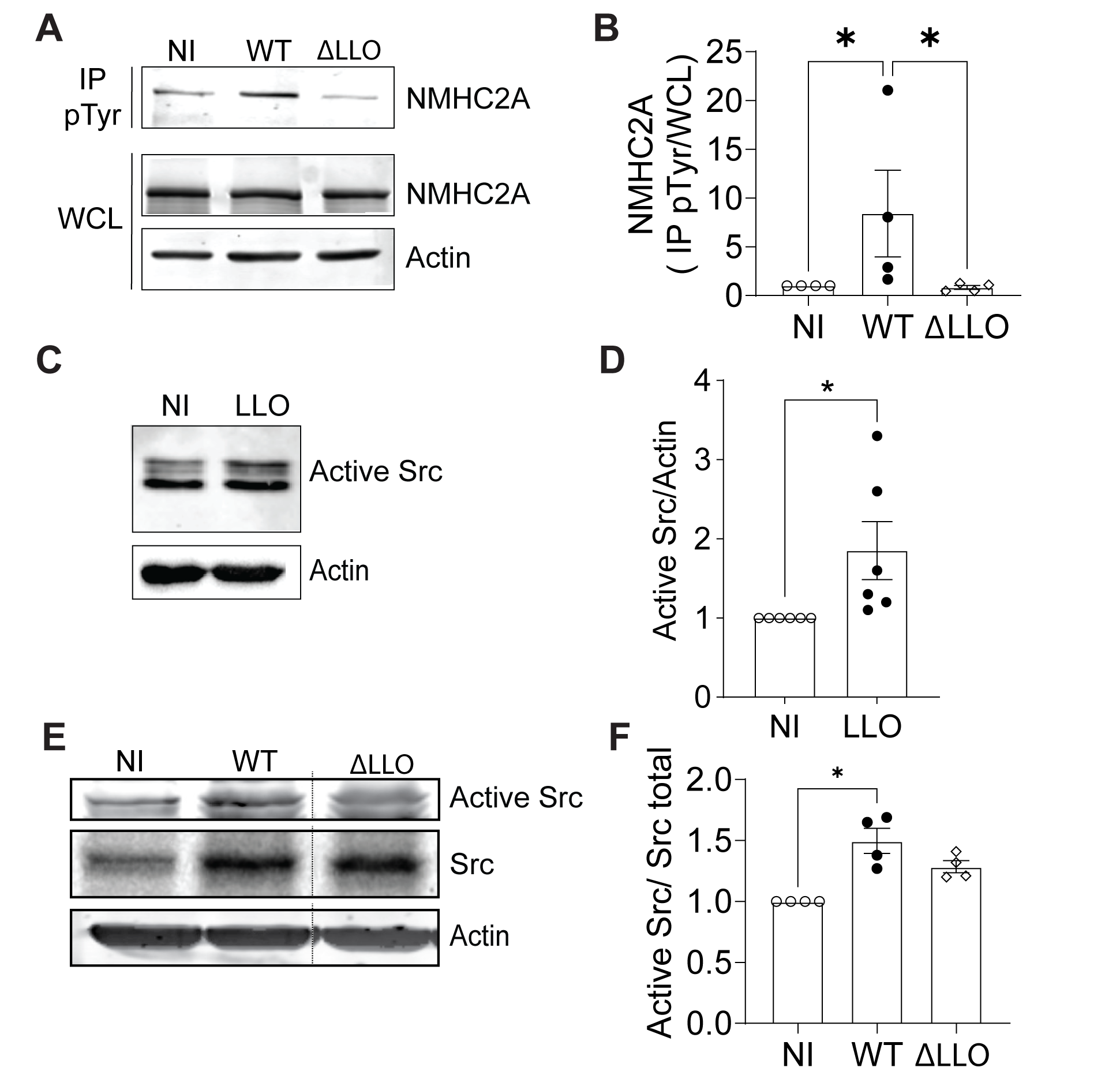

Supplement: S1 Fig — (A, B) Levels of NMHC2A measured by immunoblots on whole-cell lysates (WCL) and immunoprecipitated (IP) fractions of pTyr proteins (IP pTyr) from HeLa cells left non-infected (NI) and infected with L. monocytogenes wild-type (WT) or the isogenic strain lacking LLO expression (∆LLO) for 1 hour. Actin was used as loading control. (B) Levels of NMHC2A in the IP pTyr fraction (NMHC2A pTyr) were quantified and normalized to those detected in the WCL (NMHC2A WCL). Each dot corresponds to an independent experiment. Data correspond to mean ± SEM (n = 4); p-value was calculated using a Kruskal Wallis test with Dunn’s post hoc analysis, *p < 0.05. (C-F) Src kinase is activated by LLO in HeLa cells. (C) Immunoblot on total lysates of HeLa cells, non-intoxicated (NI) or intoxicated for 5 min with 0.5 nM LLO, showing the levels of active Src through detection of Src non-phosphorylated at Tyr530. Actin detection served as loading control. (D) Quantification of active Src (Tyr530 non-phospho Src) signals normalized to the actin levels. Each dot corresponds to a single independent experiment. Values are the mean ± SEM (n = 6); p-values were calculated using two-tailed unpaired Student’s t-test, *p < 0.05. (E) Immunoblot on total lysates of HeLa cells, non-infected (NI) or infected for 10 minutes with L. monocytogenes wild-type (WT) or the isogenic strain lacking LLO expression (∆LLO), showing the levels of active Src through detection of Src non-phosphorylated at Tyr530 and of total Src (Src). Actin detection served as loading control. (F) Quantification of active Src (Tyr530 non-phospho Src) signals normalized to the Src total levels. Each dot corresponds to a single independent experiment. Values are the mean ± SEM (n = 4); p-values were calculated using a Kruskal Wallis test with Dunn’s post hoc analysis, *p < 0.05. (TIF) [file ppat.1013945.s001.tif]

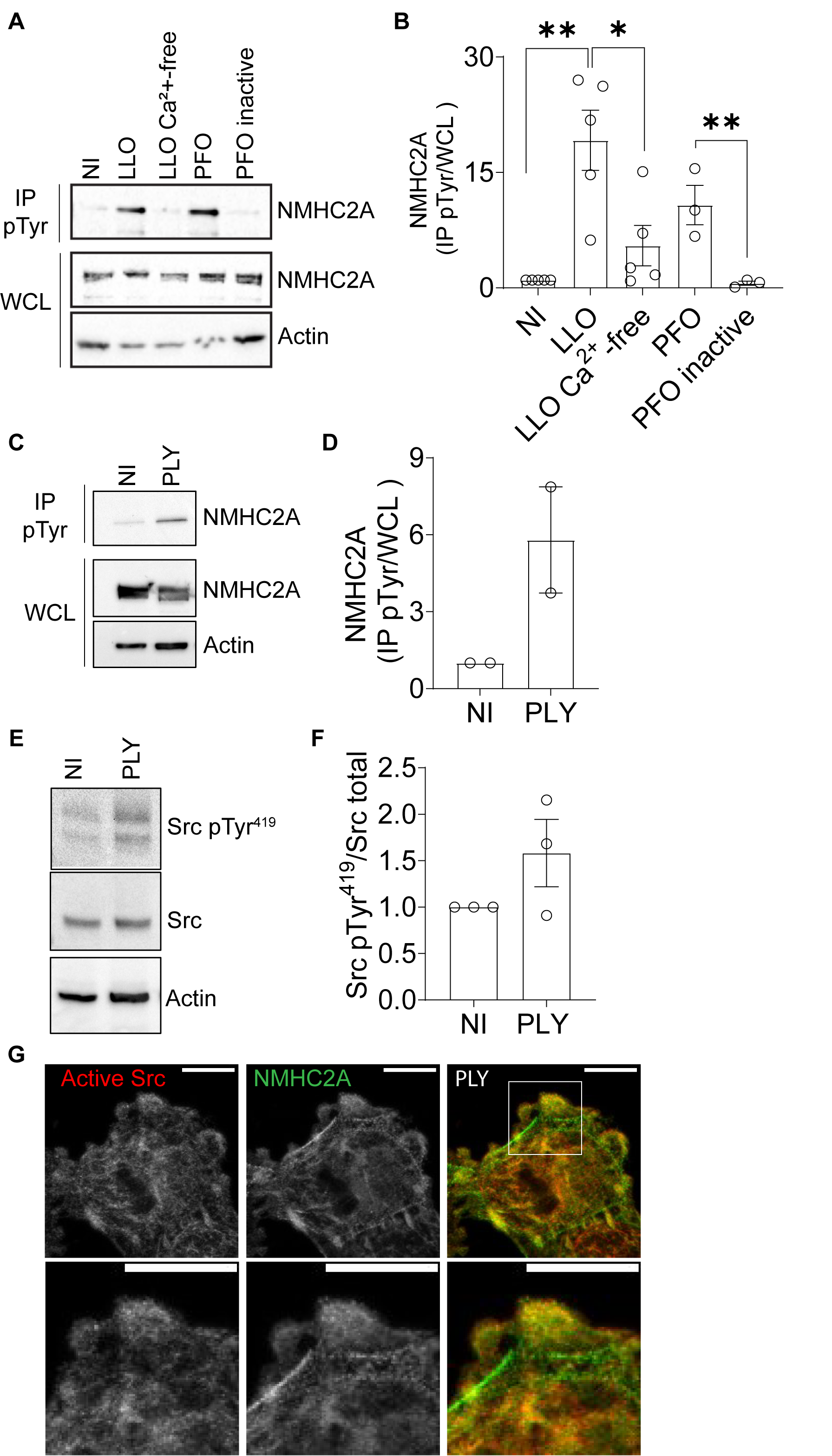

Supplement: S2 Fig — (A-D) Levels of NMHC2A measured by immunoblots on whole-cell lysates (WCL) and immunoprecipitated (IP) fractions of pTyr proteins (IP pTyr) from HeLa cells left non-intoxicated (NI) or intoxicated with LLO (0.5 nM, 10 min) in the presence (LLO) or absence of extracellular calcium (LLO Ca2+-free) or with PFO (0.5 nM, 10 min) or with PFO lacking ability to form pores (PFO inactive, 5 nM, 10 min) or with PLY (0.25 nM, 10 min). Actin was used as loading control. (B, D) Levels of NMHC2A in the IP pTyr fraction (NMHC2A pTyr) were quantified and normalized to those detected in the WCL (NMHC2A WCL). Each dot corresponds to an independent experiment. Data correspond to mean ± SEM (n = 4, n = 3 for PFO, n = 2 for PLY); p-value was calculated using a Tukey’s post hoc analysis, *p < 0.05 and **p < 0.01. (E-G) Src kinase is activated by PLY in HeLa cells. (E) Immunoblot on total lysates of HeLa cells, non-intoxicated (NI) or intoxicated with PLY (0.25 nM, 5 min), showing the levels of Tyr419-phosphorylated Src (Src pTyr419) and total Src. Actin was used as loading control. (F) Quantification of Src pTyr419 signals normalized to the levels of total Src. Each dot corresponds to an independent experiment. Values are the mean ± SEM (n = 3). (G) Confocal microscopy images of NI or PLY-intoxicated (0.25 nM, 10 min) HeLa cells, immunolabeled for active Src (red) and NMHC2A (green). Insets show PLY-induced cortical accumulations of NMHC2A enriched in active Src. Scale bar, 10 µm. (TIF) [file ppat.1013945.s002.tif]

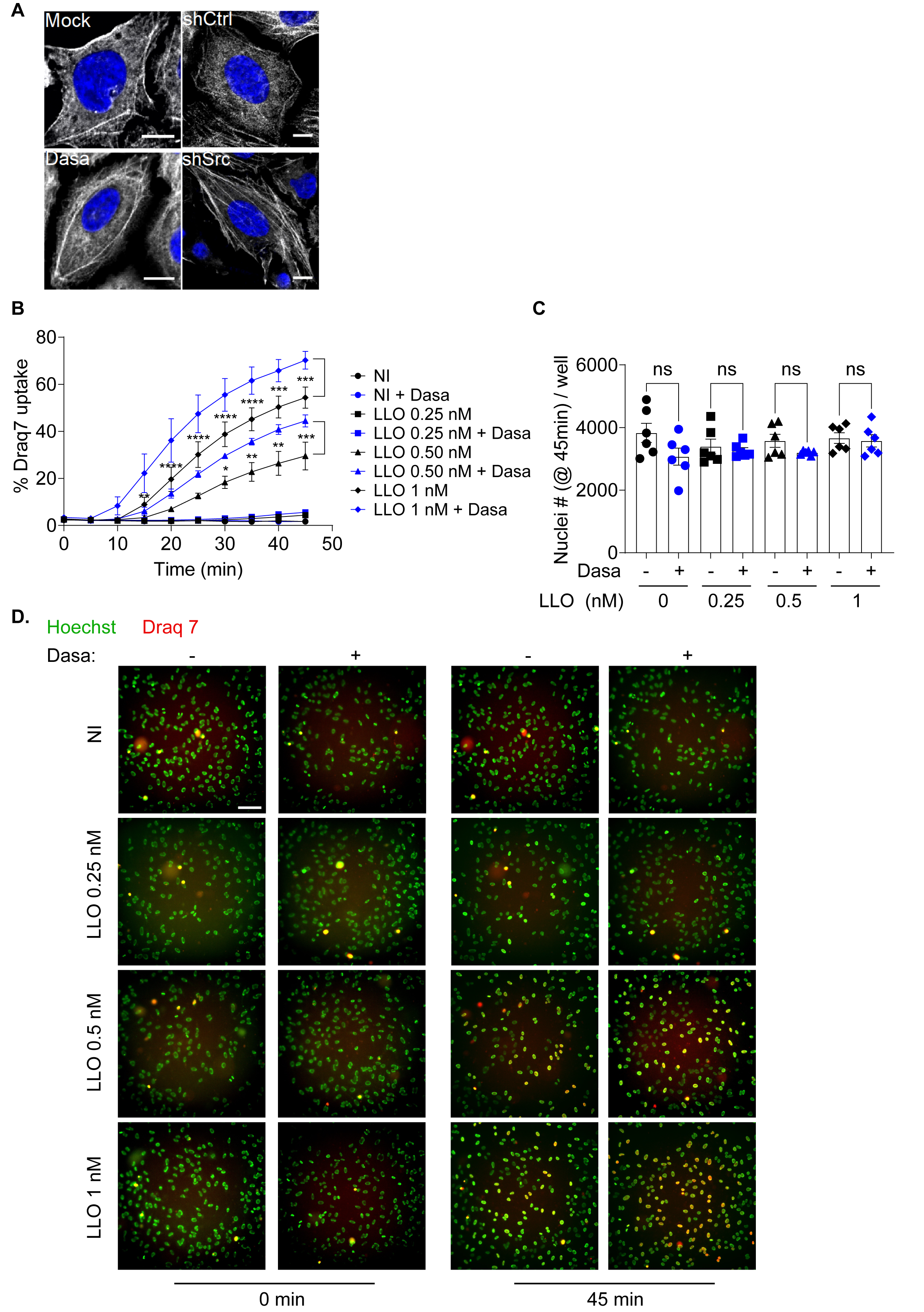

Supplement: S3 Fig — (A) Confocal microscopy images of non-intoxicated HeLa cells in control (Mock and shCtr) and Src-impaired (Dasatinib-treated and shSrc) conditions. Cells were immunolabeled for NMHC2A (greyscale) and stained with DAPI (blue). Scale bar, 10 μm. (B) Percentage of Draq7 uptake in non-intoxicated (NI) or LLO-intoxicated (0.25 nM, 0.5 nM, or 1 nM) HeLa cells in control (black) and Dasatinib-treated (blue) conditions, measured over time (every 5 min, for 45 min) by high-content microscopy. Values are the mean ± SEM (n = 2–3); p-values were calculated using two-way ANOVA with Tukey’s post hoc analysis, *p < 0.05, **p < 0.01, ***p < 0.001. (C) Average number of nuclei per well detected in each condition of (B) at 45 min. p-values were calculated using Kruskal Wallis test with Dunn’s post hoc analysis, n.s. non-statistically significant. (D) Representative high-content microscopy images of Hoechst-positive nuclei (green) and Draq7-positive nuclei (red) in each condition of (B) at 0 min and 45 min. A random field was selected. Scale bar, 100 μm. (TIF) [file ppat.1013945.s003.tif]

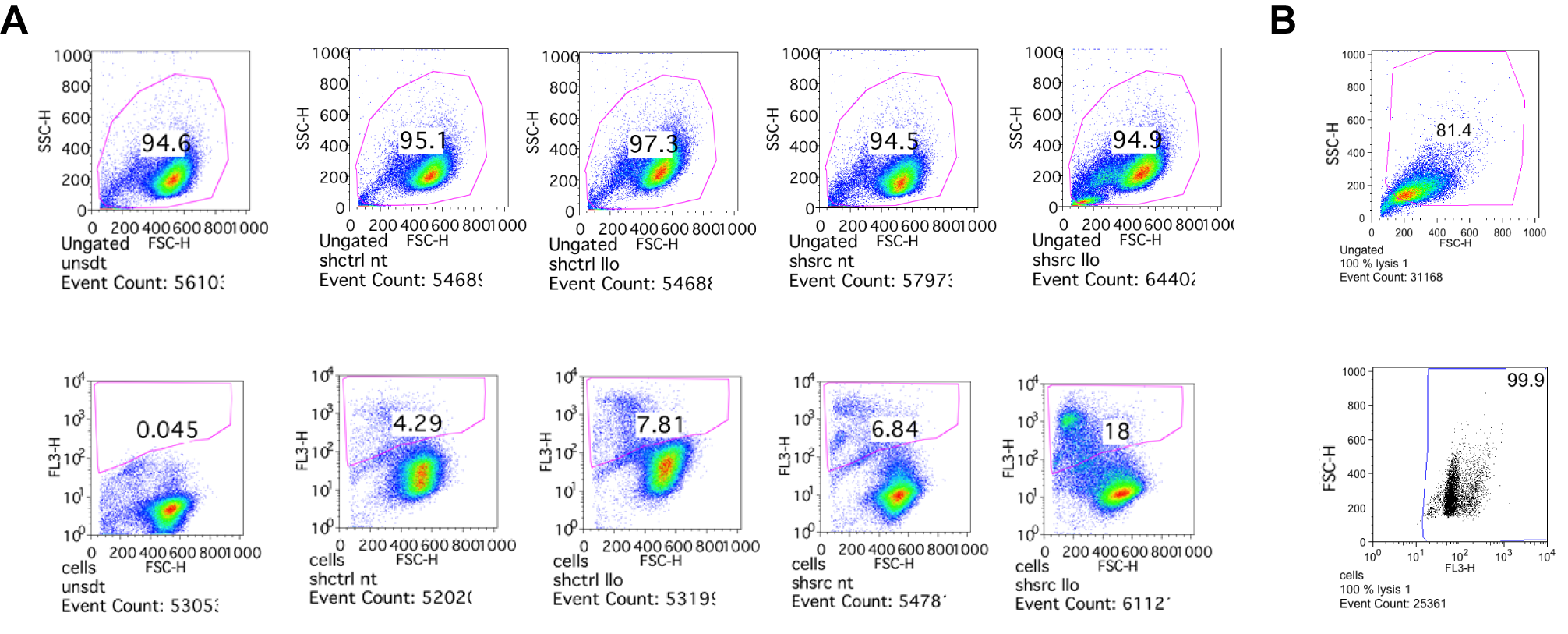

Supplement: S4 Fig — (A) Flow cytometry plots of ShCtr and ShSrc HeLa cells left non-intoxicated (nt) or intoxicated with LLO (llo). Cells were gated on forward (FSC) versus side scatter (SSC) to select the cell population (top). Next, all the subpopulations were analysed on the PI scatter (FL3) considering the unstained controls (unsdt) to establish PI-positive cells. (B) Gating strategy applied to cells treated with 0.05% Triton X-100 for 5 min to induce complete membrane permeabilization, used as a positive control for PI staining and to define the PI-positive population. (TIF) [file ppat.1013945.s004.tif]

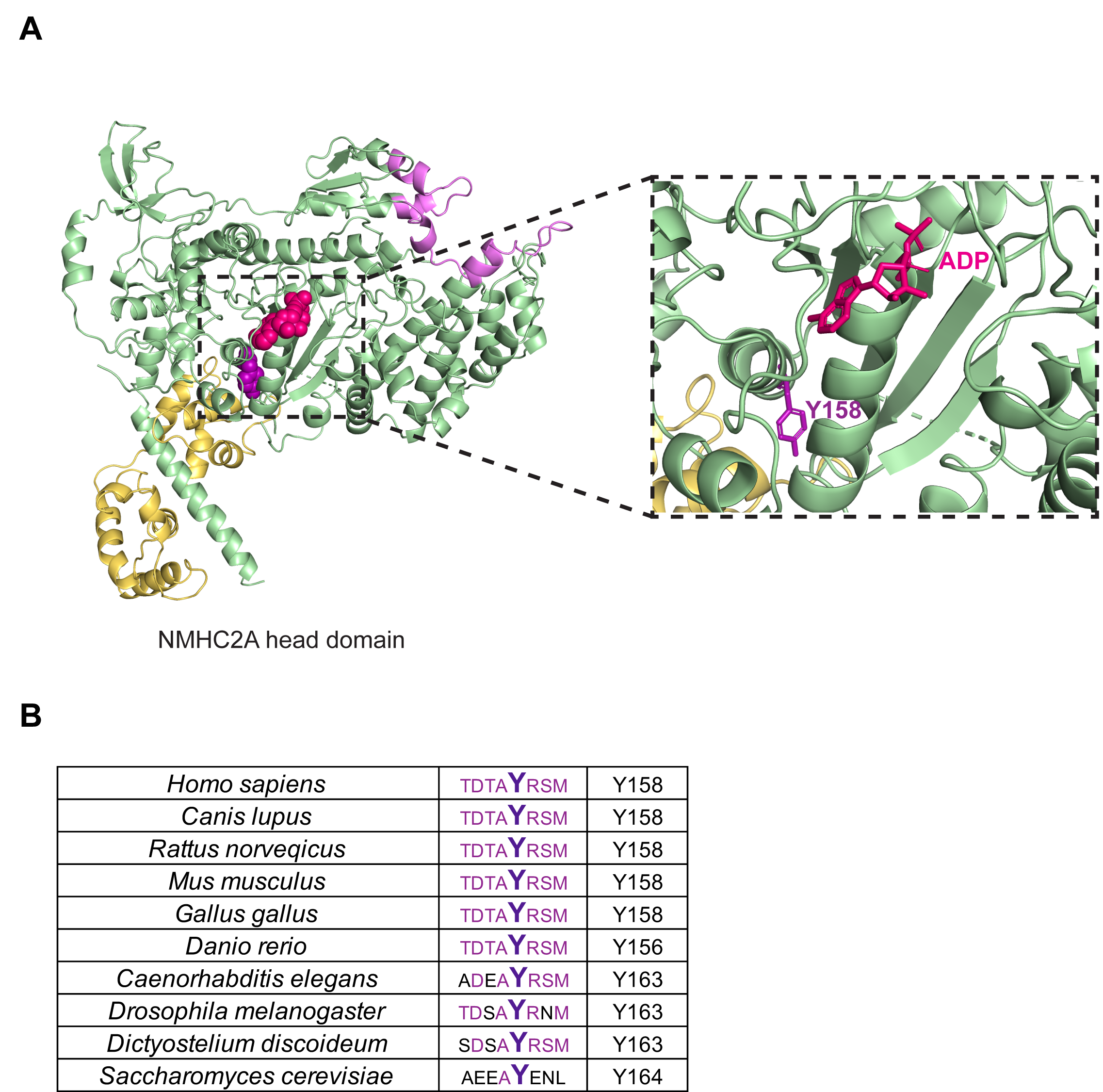

Supplement: S5 Fig — (A) Ribbon representation of the head domain of NMHC2A (green) to show Tyr158 (purple) and ADP (magenta) localization. The ELC (light yellow) and the actin-binding pocket (violet) are shown. PDB entry 1BR4. (B) NMHC2A amino-acid sequence analysis from different species and focused on the region involving the Tyr158, adapted from (17). (TIF) [file ppat.1013945.s005.tif]

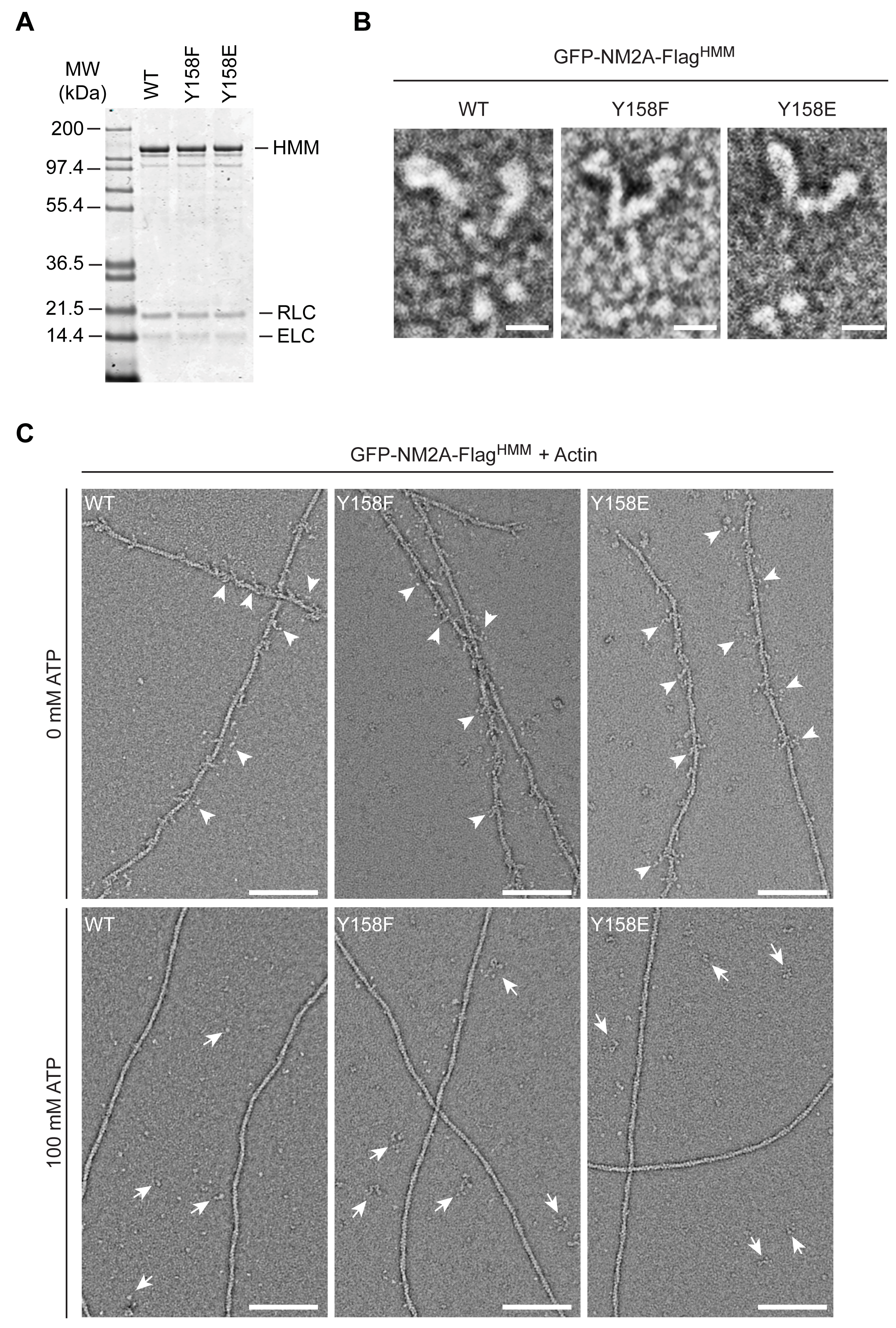

Supplement: S6 Fig — (A) SDS-polyacrylamide gel stained with Coomassie Blue showing the separation of the purified heavy mero-myosins (HMM), and the regulatory and essential light chains (RLC and ELC, respectively) for each NM2A-GFP-FlagHMM variant. (B) Representative single molecule electron microscopy images showing either a NM2A-GFP-FlagHMM-WT, Y158F or Y158E molecule. Scale bar, 10 nm. (C) Electron microscopy images showing the bound (arrow heads) and unbound (arrows) status of all purified NM2A-GFP-FlagHMM variants to actin filaments in the absence or presence of ATP. Scale bar, 100 nm. (TIF) [file ppat.1013945.s006.tif]

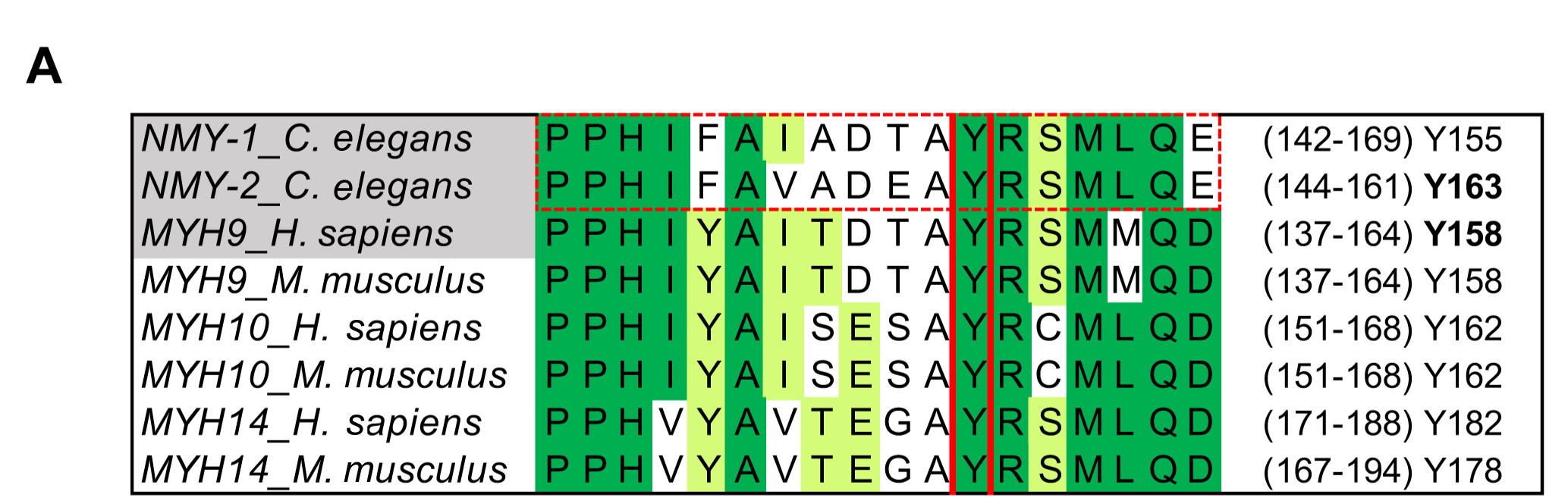

Supplement: S7 Fig — Protein sequence analysis of the NMHC2A from H. sapiens, M. musculus and C. elegans, focused in the region containing the Tyr158 in human NMHC2A. The position of the corresponding residue in the other organisms is indicated. (TIF) [file ppat.1013945.s007.tif]
