# Supplementary material for: Src-dependent tyrosine-phosphorylation of NM2A has a protective role against bacterial pore-forming toxins
Source: PLoS Pathog. 2026 Feb 23;22(2):e1013945. doi: 10.1371/journal.ppat.1013945 (PMC12959841; doi:10.1371/journal.ppat.1013945)
Supplement: S1 Table — (DOCX) [file ppat.1013945.s008.docx]

**S1 Table.** *C. elegans* strains used in this study.

| Strain | Genotype | Source |
| --- | --- | --- |
| N2 | Ancestral strain | *Caenorhabditis* Genetics Center |
| GCP693 | nmy-2 [prt143(Y163F)]I | This study |
