# Supplementary material for: Src-dependent tyrosine-phosphorylation of NM2A has a protective role against bacterial pore-forming toxins
Source: PLoS Pathog. 2026 Feb 23;22(2):e1013945. doi: 10.1371/journal.ppat.1013945 (PMC12959841; doi:10.1371/journal.ppat.1013945)
Supplement: Attachment — Submitted filename: Response to the reviewers- PloS Path.docx [file ppat.1013945.s013.docx]

**Point-by-Point response to the reviewers’ comments**

**Part I – Summary**

Please use this section to discuss strengths/weaknesses of study, novelty/significance, general execution and scholarship.

**Reviewer #1:** In this study the authors analyzed the cellular protective response to pore-forming toxins (PFTs), focusing on the role of non-muscle myosin II heavy chain A (NMHC2A) phosphorylation downstream of Src kinase activation. The authors report that Listeriolysin O (LLO) from *Listeria monocytogenes* induces Src-dependent phosphorylation of NMHC2A at tyrosine 158, which is required for cytoskeletal reorganization and plasma membrane (PM) repair but does not alter NMHC2A’s motor activity. They further demonstrate evolutionary conservation of this mechanism in Caenorhabditis elegans following exposure to Cry5Ba.
The manuscript addresses an important and timely question regarding how host cells counteract the membrane damage caused by PFTs. However, while the findings are potentially significant, several issues limit the current impact of the work and require further clarification.

We thank the reviewer for highlighting the relevance of our study on host protective responses to pore-forming toxins. We appreciate the constructive feedback and have addressed the raised points in detail below.

1. The novelty of the work is not sufficiently highlighted. LLO-induced Src activation and NMHC2A phosphorylation during bacterial infection have both been reported (Sousa et al., 2007; Almeida et al., 2015). The authors should explicitly state what is new here: Is it the identification of Tyr158 as the relevant residue? The functional dissection separating NMHC2A’s motor activity from its role in membrane repair? Or the demonstration of evolutionary conservation using Cry5Ba?

We thank the reviewer for this helpful comment. We would like to clarify that neither LLO-induced Src activation nor LLO-driven NMHC2A phosphorylation had been previously reported. [Sousa et al. (2007)](https://doi.org/10.1111/j.1462-5822.2007.00984.x) described Src activation during *Listeria* infection, but its dependency on LLO was not established. Likewise, [Almeida et al. (2015)](https://doi.org/10.1074/jbc.M114.591313) reported NMHC2A tyrosine phosphorylation in response to *Listeria* infection, but the contribution of LLO to this modification was unknown. The key novelties of our work are therefore:

- The demonstration that both Src activation and NMHC2A Tyr158 phosphorylation are specifically triggered by LLO.
- The finding that other pore-forming toxins (PFTs) related to LLO (e.g. PLY and PFO) also trigger the NMHC2A Tyr158 phosphorylation in a pore- and calcium-dependent manner, suggesting that this phosphorylation might be a conserved response against PFT-producing bacteria.
- The functional characterization of NMHC2A in the context of LLO intoxication, showing that its role is independent from its canonical motor activity.
- The *C. elegans* data indicate potential conservation of related protective mechanisms, as mutants in the phosphorylation site of the NMHC2A homolog display enhanced sensitivity to Cry5Ba, LLO, and PFO.

We have revised the manuscript to better highlight these points (Page 11, First paragraph of the Discussion and Page 2, Abstract).

2.The cross-species experiment with C. elegans and Cry5Ba is potentially interesting, but its mechanistic relevance remains unclear. How directly comparable are these systems? Does Cry5Ba also activate Src or a functional homolog in nematodes?, and does it target NMHC2A orthologs in a similar manner? The evolutionary conservation claim should be either substantiated with mechanistic data or substantially toned down.

We appreciate the reviewer’s insightful comment regarding the mechanistic interpretation and evolutionary relevance of our *C. elegans* experiments. A key limitation is that we currently lack validated tools to directly monitor Src or Src-like kinase activation in *C. elegans* during PFT exposure. *C. elegans* encodes Src-family kinases (SRC-1 and SRC-2) that are well-established regulators of cytoskeletal dynamics. SRC-1 is a non-receptor tyrosine kinase structurally and functionally related to mammalian Src. It has been shown to control actin-dependent processes such as cell migration, growth cone protrusion, and polarity through phosphorylation-dependent signaling pathways ([Bei et al., 2002](https://www.sciencedirect.com/science/article/pii/S1534580702001855); [Itoh et al., 2005](https://journals.biologists.com/dev/article/132/23/5161/43153/SRC-1-a-non-receptor-type-of-protein-tyrosine); [Liu et al., 2010](https://www.sciencedirect.com/science/article/pii/S0012160609014663); [Mahadik et al., 2024](https://doi.org/10.1371/journal.pone.0295701)). These roles underscore that Src-like signaling exists in nematodes and can modulate actomyosin-associated cellular behaviors, even though its involvement in PFT responses has not been directly demonstrated. Importantly, complete deletion of the *src-1* locus results in viable but sterile adults ([Mahadik et al., 2024](https://doi.org/10.1371/journal.pone.0295701)), reflecting essential roles in development and limiting its utility for infection-based mechanistic dissection.

Our goal in using *C. elegans* was not to claim direct conservation of an upstream Src–NMHC2A signaling axis, but rather to test whether tyrosine phosphorylation of the myosin II heavy chain itself (NMY-2) contributes to organismal resistance to PFTs. The *C. elegans-*Cry5Ba intoxication system is a well-established *in vivo* model for dissecting host defense pathways against PFTs ([Bellier et al., 2009](https://doi.org/10.1371/journal.ppat.1000689); [Bischof et al., 2008](https://doi.org/10.1371/journal.ppat.1000176); [Chen et al., 2010](https://doi.org/10.1371/journal.pone.0009494); [Huffman et al., 2004](https://doi.org/10.1073/pnas.0404073101)). Motivated by the strong track record of this model, we examined the susceptibility of *nmy-2* Y163F mutant animals to Cry5Ba. In the revised manuscript, we now provide new data showing that *C. elegans* Y163F animals exhibit increased sensitivity to purified LLO and PFO (but not to purified “PFO inactive”, unable to form pores) in soaking assays (new data in revised Figure 5 B, C, D). These results support a conserved functional requirement for NMHC2A/NMY-2 tyrosine phosphorylation in the cellular response to multiple PFTs.

To avoid overinterpretation, we have revised the Results and Discussion section (pages 11 and 15, respectively) to explicitly state that we do not demonstrate activation of Src or Src-like kinases in *C. elegans* and that our conclusions are limited to the functional importance of the NMHC2A/NMY-2 tyrosine phosphorylation in PFT resistance. We believe this appropriately tones down evolutionary claims while preserving the value of the cross-species findings.

**Reviewer #2:** In “Src-dependent tyrosine-phosphorylation of NM2A has a protective role against bacterial pore-forming toxins,” Brito et al identify a host protective pathway triggered by the addition of purified exogenous LLO where tyrosine kinase Src is activated, leading to phosphorylation of NMHC2A at tyrosine 158, that is critical for cytoskeletal reorganization and PM repair. The authors also use C. elegans to show that phosphorylation of a NMHC2A homolog at the conserved tyrosine is required for survival under heat shock and after the organisms consume E. coli expressing a pore-forming toxin. This work shows a link between host sensing of PM damage and actomyosin remodeling.

We thank the reviewer for recognizing the relevance of our study on Src-dependent cytoskeletal responses to pore-forming toxins, as well as the conserved protective mechanism observed in *C. elegans*. We appreciate the positive assessment of our work.

**Reviewer #3:** In this manuscript by Brito, Mesquita, Pereira et al., the authors propose a host signaling pathway – through Src kinase and non-muscle heavy chain myosin 2A (NMHC2A) – that mobilizes cytoskeletal remodeling in the host to maintain plasma membrane integrity in response to damage from pore-forming toxins.

We sincerely thank the reviewer for the time and thoughtful consideration given to our manuscript. We greatly appreciate the constructive feedback and have addressed all comments in detail below.

The authors first provide thorough evidence that NMHC2A and Src are both post-translationally modified after exposure to the pore-forming toxin, LLO. This results in the activation and re-localization of Src to the periphery of the cell – corresponding to where LLO-induced damages would be incurred, although importantly the authors never directly localize Src signaling to sites of membrane damage.

In our previous work ([Mesquita et al., 2017](https://www.embopress.org/doi/full/10.15252/embr.201642833)), we have demonstrated that cortical sites where NMHC2A accumulates in response to LLO intoxication or *L. monocytogenes* infection correspond to membrane damage sites. Specifically, NMHC2A accumulation coincides with exofacial phosphatidylserine exposure, a well-established marker of plasma membrane injury ([Czuczman et al., 2014](https://www.nature.com/articles/nature13168)).

In this study, we extend these findings by showing that active Src is recruited to the same cortical regions where NMHC2A accumulates following LLO treatment (Figure 1H). Because NMHC2A accumulation marks sites of LLO-induced membrane damage, our data demonstrate that Src signaling occurs precisely at damaged plasma membrane sites.

In the revised manuscript, we now explicitly state (Page 7) that NMHC2A cortical accumulation serves as a hallmark of plasma membrane damage sites, thereby clarifying the localization of Src signaling relative to LLO-induced injury.

Next, the authors demonstrate that Src is important for NMHC2A phosphorylation using various strategies spanning from the use of chemical inhibitors, knockdown of Src, as well as the use of constitutively active and kinase dead variants that cause dramatic phenotypes regarding the phosphorylation status of NMHC2A. The diversity of approaches used by the authors as well as the effect sizes of the quantifications presented (with exception of Figure 2J, please see below) nicely strengthen the claims made in the first two figures.

We thank the reviewer for this positive comment. As suggested, we have repeated the quantifications shown in Figure 2J; please see our response in the “Minor points” section.

The authors also provide thorough biochemical data using purified variants of phosphomimetic and phosphoablative point mutations to support the claim that the phosphorylation status of NMHC2A does not affect its kinetic properties with regards to ATPase activity nor its ability to associate with co-sedimentation with actin. Finally, the authors finish their manuscript with data supporting their claim that the phosphorylation status of the homologous protein in C. elegans is necessary for survival against the Cry5B pore-forming toxin and heat stress and extrapolate this finding to suggest that this is due to defects in membrane repair although membrane repair is never directly addressed in this model.

We agree with the reviewer’s assessment. Because we did not directly measure plasma membrane damage or repair in *C. elegans*, attributing the observed phenotypes to defects in membrane repair was indeed an overinterpretation. In the revised manuscript (pages 11 and 12), we have removed this mechanistic extrapolation and now state that the phosphorylation status of NMY-2 is required for resistance to PFT exposure and heat stress, without assigning a specific mechanism. We explicitly note that the up and downstream processes responsible for this phenotype remain to be determined (page 14).

Despite strong validation of Src-NMHC2A signaling in the presence of LLO, multiple of the major conclusions of this manuscript are incompletely supported. One major conclusion is that pore forming toxins trigger this host cell response. This group had previously demonstrated that Listeria and E. coli infection (no pore forming toxin) can both trigger the response but the investigators never determined if Listeria’s induction was dependent on expression of LLO using an hly mutant.

We agree with the reviewer. In the revised version of our manuscript, we now present new infection experiments showing that while the wild-type *L. monocytogenes* strain induces activation of Src and tyrosine phosphorylation of NMHC2A, the ∆LLO mutant fails to do so (Figure S1A, B, E, F). These results confirm that triggering of the Src-NMHC2A signalling axis depends on LLO expression during infection.

Similarly, while the investigators used purified LLO in this study, they did not have a pore formation deficient mutant and did not demonstrate that the signaling cascade is a broadly conserved response using another purified toxin in their robust *in vitro* signaling assays.

In the revised version of the manuscript, we now include new experiments that directly address the reviewer’s concern. We tested additional PFTs, including PLY (*Streptococcus pneumoniae*) and PFO (*Clostridium perfringens*), and found that, similar to LLO, both toxins induce tyrosine phosphorylation of NMHC2A (Figure S2A-D). PLY also reveals to activate Src kinase (Figure S2E, F, G).

We further demonstrate that NMHC2A phosphorylation depends on pore formation and the resulting calcium influx, as the phosphorylation event was not observed when cells were intoxicated with a pore-deficient PFO mutant or with LLO in calcium-free medium (Figure S2A, B).

Finally, the claim that this signaling facilitates membrane repair is not supported as the authors never determine if defects in signaling result in host cell death due to inability to repair the pores induced by LLO but rather they simply measure pore formation by PI which should not be different based on their hypothesis but rather the repair of these pores should be. Is it possible that disruptions in these pathways result in altered cholesterol concentrations leading to differential activity of LLO? Additionally, in this assay the shCtr and the Dasa mock are significantly different from one another even though they should be equivalent controls, thus making it difficult to believe that the differences in PI staining in the experimental samples are indeed biologically different.

We thank the reviewer for these thoughtful comments and the opportunity to clarify our experimental design and interpretation.

**Sub-lytic toxin concentrations and absence of cell death:** We agree that our manuscript did not explicitly state an important point: all experiments were performed using *sub-lytic* concentrations of LLO. At these concentrations, cells are not driven to cell death but instead are able to sense the damage, activate repair pathways, and recover membrane integrity ([Alves, Pereira et al., 2022](https://www.mdpi.com/2072-6651/15/1/4)). We determined the sub-lytic range for each toxin batch and confirmed that cells exposed to these doses are capable of membrane repair after toxin washout experiments. Thus, our assays do not monitor lethality but rather the signaling and damage response under non-lethal pore-forming conditions (Figure S3B, C). We now state that we are using sub-lytic doses in the revised version of our manuscript (Page 6).

**Difficulty of temporally separating pore formation (damage) from repair:** We fully agree that measuring pore formation and repair independently is technically challenging. Membrane repair is known to be an extremely rapid process, occurring within seconds to a few minutes after injury. Because our intoxication periods (10 min) are substantially longer than the rapid onset of repair, the readouts necessarily reflect a combination of damage and repair. In addition, monomer binding, oligomerization, and pore formation do not occur synchronously across the cell population; therefore, at any given time point, some pores will already have been repaired while others are newly formed. For these reasons, assays designed to measure “only damage” are difficult to interpret in this context, and PI uptake provides a functional integrated measure of injury and repair. As we did not perform toxin washout experiments in this study, we agree that our interpretations should be restricted to describing the net level of damage, rather than attributing the observed effects solely to differences in repair efficiency. This has been corrected in the revised version of the manuscript (Page 9).

**Potential effects of Src pathway disruption on membrane cholesterol:** We acknowledge that we cannot fully exclude the possibility that perturbation of Src signaling alters plasma membrane cholesterol levels. However, because LLO exhibits increased activity in cholesterol-rich membranes, enhanced membrane damage would require a corresponding increase in membrane cholesterol. To our knowledge, there is no evidence that Src inhibition increases cholesterol abundance in a way that would sensitize cells to LLO. Although subtle changes in lipid composition cannot be ruled out, we consider this explanation unlikely. Instead, our data are more consistent with a defect in signaling-dependent cell response to membrane damage rather than with altered toxin activity.

**Difference between shCtrl and Dasa-mock controls:** We appreciate the reviewer’s concern regarding the control conditions. From our analysis of images shown in upper left and bottom left panels of Figure 3A, the shCtrl and mock (referred as Dasa mock by the reviewer) samples are comparable and fall within the expected range of biological variability for independent cell preparations and imaging fields. In addition, we didn’t notice any significant differences in the other analysis done (e.g. number and distribution of NMHC2A accumulations, PI permeability assays; Figure 3B-E). Therefore, the minor variations between the two controls (shCtrl and Mock) do not affect our interpretation that inhibition of Src signaling impacts the cellular response to pore-forming toxins.

Another major conclusion is that the phosphorylation does not affect the biochemical activity but does affect the response to LLO, however given that the point mutants utilized in the biochemical assays are never expressed in the cell culture models it is impossible to know if these mutations actually affect function in the cells either. It is possible that they affect protein protein interactions in their functional molecular complexes in the cell and this is not recapitulated in the *in vitro* biochemical system, but as they are never tested in the cell the impact of the biochemical characterization is unclear.

The same point mutations used in biochemical assays in the present study were already characterized in cells in our previously published manuscript ([Brito et al, 2023](https://www.mdpi.com/2073-4409/12/14/1871)). In that study, the Tyr158 point mutations were introduced into NMHC2A fused with GFP and overexpressed in cells. Despite the inherent limitations of overexpression experiments, we showed that the phosphorylation status of Tyr158 defines cytoskeletal organization, affects the assembly/disassembly of focal adhesions, and interferes with cell migration. Cells overexpressing a non-phosphorylatable NM2A variant display increased stress fibers and larger focal adhesions. The lack of Tyr158 phosphorylation increases NMHC2A turnover, thereby altering its dynamics within assembled NM2A filaments. We didn’t find any evidence of changes in protein–protein interactions.

Furthermore, the viability of the *C. elegans* non-phosphorylatable mutant (this study) suggests that this mutation does not interfere with major protein–protein interactions required for NM2A function.

In sum, the experiments presented in this manuscript do a good job of dissecting this signaling pathway, demonstrating the biochemical impact of specific point mutations in NMHC2A and the impact of these mutations in a C. elegans model. However, the authors fall short of demonstrating that it is pore formation that triggers these responses, that the outcome of the response is membrane repair to protect from pore forming toxins and that the phosphomimetic and ablative mutations they studied biochemically have phenotypes in cellulo. Specific recommendations to address these concerns are:

**Part II – Major Issues: Key Experiments Required for Acceptance**
Please use this section to detail the key new experiments or modifications of existing experiments that should be absolutely required to validate study conclusions.
Generally, there should be no more than 3 such required experiments or major modifications for a "Major Revision" recommendation. If more than 3 experiments are necessary to validate the study conclusions, then you are encouraged to recommend "Reject".

**Reviewer #1:**

1. Mechanistic depth and spatial dynamics
The central claim is that NMHC2A phosphorylation at Tyr158 is essential for cytoskeletal remodeling and PM protection. However, the study provides limited insight into how this modification coordinates actomyosin activity at specific sites of toxin-induced damage. Does phosphorylation regulate NMHC2A recruitment, assembly, or interaction with accessory proteins at PM lesions? Without this spatial and mechanistic resolution, the functional model remains incomplete.

We agree with the reviewer that the data presented here do not fully resolve the spatial and temporal regulation of NMHC2A during PM repair. Nevertheless, when considered together with our previously published findings, our data provide strong mechanistic support for the conclusion that phosphorylation of NMHC2A at Y158 is critical for the cytoskeletal remodeling that enables efficient PM responses following pore-forming toxin challenge.

We previously demonstrated ([Mesquita et al, 2017](https://www.embopress.org/doi/full/10.15252/embr.201642833)) that LLO-induced PM damage triggers the recruitment and accumulation of NMHC2A to the cell cortex at sites containing exofacial phosphatidylserine, a hallmark of PM damage. In addition, we found that NMHC2A cortical accumulation is transient, associated with PM blebbing and required for effective PM repair.

Here we report that at least part of the cortical accumulated NMHC2A is phosphorylated in Y158 (Figure 2G,H). As we previously showed for *Listeria monocytogenes* infection ([Almeida et al., 2015](https://pmc.ncbi.nlm.nih.gov/articles/PMC4375491/)), this phosphorylation is mediated by Src kinase (Figure 2A-F). Importantly, in Src-defective cells, NMHC2A cortical accumulations are still detected in response to LLO (Figure 3A-D), indicating that NMHC2A tyrosine phosphorylation per se is not required for NMHC2A cortical accumulation.

Finally, NMHC2A ATPase activity and its ability to bind actin are not affected by the phosphorylation status of Y158 (Figure 4). However, our previous data ([Brito et al, 2023](https://www.mdpi.com/2073-4409/12/14/1871)) indicate that the lack of Y158 phosphorylation increases the NMHC2A turnover thus affecting its dynamics within assembled NM2A filaments.

Altogether our data thus indicate that phosphorylation at Y158 does not regulate NMHC2A recruitment to PM lesions but rather modulates its dynamics at these cortical sites. We therefore propose that this phosphorylation fine-tunes NMHC2A filament turnover and actomyosin remodeling locally at damage sites, promoting the transient cytoskeletal reorganization necessary for PM repair. Although the precise molecular partners affected by this modification remain to be identified, our work provides a mechanistic basis linking Src-dependent phosphorylation with spatially restricted NMHC2A activity during membrane damage response.

2. Focus on a single phosphorylation site
By restricting analysis to Tyr158, the authors may be overlooking additional phosphorylation events or post-translational modifications that could also regulate NMHC2A. Given the complexity of Src signaling and cytoskeletal dynamics, the exclusive focus on one site appears narrow. At minimum, a broader phosphoproteomic analysis should be discussed, and ideally preliminary evidence provided.

We agree with the reviewer that NMHC2A function in response to LLO pore formation may also be regulated by post-translational modifications other than phosphorylation of Y158. Our interest in tyrosine phosphorylation signalling originated from our finding that *Listeria* infection activates Src kinase ([Sousa et al, 2007](https://onlinelibrary.wiley.com/doi/10.1111/j.1462-5822.2007.00984.x)) and that NMHC2A is a tyrosine kinase substrate during infection by multiple human pathogens ([Almeida et al, 2015](https://www.sciencedirect.com/science/article/pii/S0021925820639813?via%3Dihub)).

We performed several phosphoproteomic analyses of NMHC2A under *Listeria*-infected and LLO-intoxicated conditions (compared with non-infected or non-intoxicated controls). In all cases, Y158 was the only tyrosine found to be phosphorylated, which provides a strong experimental rationale for our focus on this site.

Notably, NMHC2A has been recently shown to be phosphorylated on distinct tyrosine residues. Viral infection drives phosphorylation of tyrosine residues Y277 and Y1805 ([Ansin et al., 2025)](https://doi.org/10.1128/jvi.00241-25), a process mediated by Src kinases, which promotes cortical NMHCIIA accumulation (included in the Discussion, page 14). These findings underscore that different pathogens can exploit distinct NMHC2A tyrosine phosphorylation sites. Our data therefore suggest that Y158 represents the dominant tyrosine phosphorylation event associated with PFT–induced membrane damage, rather than excluding the importance of other sites in different contexts.

In addition to tyrosine phosphorylation, our phosphoproteomic analyses also identified serine phosphorylation of NMHC2A, notably S1943, which is located in the NMHC2A C-terminal region. Phosphorylation of S1943 by CK2 is well characterized and inhibits NM2A filament assembly by modulating binding of the metastasis-associated protein S100A4 ([Dulyaninova et al, 2005](https://pubs.acs.org/doi/10.1021/bi0500776); [Li et al, 2003](https://pubs.acs.org/doi/10.1021/bi0354379)). Although pS1943 was not addressed experimentally here, we have independent evidence implicating S100A4 in membrane damage responses following pneumolysin-induced damage (Pereira et al., manuscript in preparation).

To provide initial validation of the phosphoproteomic data, we performed a preliminary experiment in which cells were left untreated or intoxicated with sub-lytic doses of LLO (0.5 nM). Phospho-serine–containing proteins were immunoprecipitated using an anti-phosphoserine antibody and probed for NMHC2A. NMHC2A was enriched in the immunoprecipitated fraction from LLO-treated cells, while total NMHC2A levels in whole-cell lysates (WCL) were comparable between untreated and intoxicated conditions. Although based on a single experiment and requiring further confirmation, these results are consistent with LLO-induced serine phosphorylation of NMHC2A (see figure below).

We now explicitly discuss the potential contribution of additional phosphorylation events, including serine and alternative tyrosine residues, to NMHC2A regulation in response to pore-forming toxins in the revised manuscript (page 14).

**Reviewer #2:**

This manuscript is clearly written and straight forward, however it is unclear whether activation of NM2A though Src is a mechanism that occurs in response to a true bacterial infection. The authors expose cell to exogenous LLO from a facultative intracellular pathogen. Typically, LLO is critical for the generation phagosomal pores, although eukaryotic cells may potentially experience a very limited amount of LLO at the PM; it is unclear whether the amounts used here are biologically relevant.

We appreciate the reviewer’s insightful comment regarding the physiological relevance of NM2A activation through Src in response to LLO exposure.

We previously reported the Src-dependent phosphorylation of NMHC2A in epithelial cell lines infected with *Listeria monocytogenes* ([Almeida et al, 2015](https://pmc.ncbi.nlm.nih.gov/articles/PMC4375491/)), although the bacterial factor driving this modification was not identified. In the same study, we also showed that NMHC2A tyrosine phosphorylation also occurs in response to other bacterial pathogens such as EPEC, EHEC and *E. coli* expressing the invasin from *Yersinia pseudotuberculosis*, thus indicating that this signaling event is a general host response to bacterial infection.

As noted by the reviewer, *Listeria monocytogenes* is a facultative intracellular pathogen but it can also exist extracellularly, namely in the bloodstream during infection. Therefore, LLO secreted to the extracellular milieu could indeed interact with host cell membranes. Consistent with this, previous studies have shown that LLO damages the PM from the extracellular milieu at a neutral pH ([Bavdek et al., 2007](https://pubs.acs.org/doi/10.1021/bi602497g)), and contributes to the entry of *L. monocytogenes* into epithelial cells ([Vadia et al, 2011](https://doi.org/10.1371/journal.ppat.1002356)).

To further support this point, in the revised manuscript we include data from infection experiments showing that while the wild-type *L. monocytogenes* strain induces activation of Src and tyrosine phosphorylation of NMHC2A, the ∆LLO mutant fails to do so (Figure S1A, B, E, F). Thus confirming that triggering of the Src-NMHC2A signalling axis depends on LLO expression during infection. We also include data using PFTs produced by extracellular bacteria (e.g., pneumolysin and perfringolysin O, PLY and PFO respectively) (Figure S2), which similarly to LLO induce NMHC2A phosphorylation. These results reinforce that the Src-dependent phosphorylation of NMHC2A that we observe is not restricted to LLO or to intracellular pathogens, but reflects a general host response to pore formation.

Finally, we acknowledge the reviewer’s concern regarding the biological relevance of the LLO concentrations used. While this point is inherently difficult to address directly, we would like to emphasize that all our experiments employ sub-lytic doses of LLO (Stated in the revised version, page 6). Importantly, the effects observed with purified LLO are recapitulated when cells are infected with wild-type *L. monocytogenes*, supporting the physiological relevance of our findings. We also note that the concentration used in our study (0.5 nM) is substantially lower than those reported in several published studies, which can reach up to 6 nM ([Hamon et al., 2007](https://www.pnas.org/doi/full/10.1073/pnas.0702729104); [Hamon et al., 2011](https://journals.asm.org/doi/10.1128/iai.01243-10); [Stavru et al., 2011](https://www.pnas.org/doi/10.1073/pnas.1100126108); [Stavru et el., 2013](https://doi.org/10.1073/pnas.1315784110)).

Therefore, the mechanism of NM2A activation by PM pore formation may be a general mechanism to perturbation of the membrane or a change in membrane fluidity which is interesting but not necessarily a response to bacterial pathogenesis. These experiments would be more plausible as a response to bacterial infection, if a pore-forming toxin from a generally extracellular bacterium were used, for example PLY from *Streptococcus pneumoniae* or SLO from *Streptococcus pyogenes*.

To directly address this concern, we performed additional experiments using PFTs from extracellular bacteria, including PLY (*Streptococcus pneumoniae*) and PFO (*Clostridium perfringens*). Both PFTs tested triggered the tyrosine phosphorylation of NMHC2A (Figure S2A-D). We further show that this event depends on pore formation and the consequent influx of calcium, as NMHC2A phosphorylation was impaired in cells intoxicated with a pore-deficient PFO variant, or with LLO in calcium-free medium (Figure S2A, B). These findings indicate that NMHC2A tyrosine phosphorylation represents a conserved host response to toxin-induced pore formation.

As mentioned before, the revised manuscript includes infection-experiment data showing that the wild-type *L. monocytogenes* activates Src and induces NMHC2A tyrosine phosphorylation, whereas the ∆LLO mutant does not (Figure S1A, B, E, F), confirming that activation of the Src–NMHC2A axis is responding to infection.

As presented, LLO and the *Bacillus thuringiensis* homolog are used as tools to induce PM pore formation but whether the identified NM2A activation pathway happens in the context of a true bacterial infection and not the addition of exogenous protein is unclear. Can the authors show evidence that NM2A activation happens in the context of *Listeria monocytogenes* infection of host cells or infections of C. elegans with Bacillus thuringiensis? Or does PLY or SLO trigger the same response observed from LLO?

To address the reviewer’s concern, we provide both previously published and new evidence. In our previous study ([Almeida et al, 2015](https://pmc.ncbi.nlm.nih.gov/articles/PMC4375491/)) we demonstrated that NMHC2A tyrosine phosphorylation occurs during cellular infection by *L. monocytogenes*, EPEC, EHEC and an *E. coli* strain expressing the invasin from *Yersinia* *pseudotuberculosis*. We now performed cellular infections with *L. monocytogenes* wild-type and the isogenic strain ∆LLO. In addition, in [Mesquita et al, 2017](https://www.embopress.org/doi/full/10.15252/embr.201642833), we showed that NMHC2A accumulates at the cell cortex at sites of PM damage in response to both purified LLO and infection with wild-type *L. monocytogenes*, and not with the ∆LLO bacterial mutant, demonstrating that LLO is required to trigger NMHC2A response.

In the revised version of our manuscript, we now present new infection experiments showing that while the wild-type *L. monocytogenes* strain induces activation of Src and tyrosine phosphorylation of NMHC2A, the ∆LLO mutant fails to do so (Figure S1A, B, E, F). These results confirm that triggering of the Src-NMHC2A signalling axis depends on LLO expression during infection. Furthermore, we show that PLY and PFO (Figure S2A-D) elicit the same response as LLO, suggesting that NMHC2A tyrosine phosphorylation represents a conserved host response to pore-forming bacterial toxins.

**Reviewer #3:**

1. If the authors opt to maintain their argument that this Src-NMHC2A pathway is a response to LLO-induced damage; thus, informing us of host responses to Listeria pathogenesis, the authors should strongly consider repeating Figures 1AB or 1CD using host cells that were either not infected or infected with WT or Δhly Listeria. Furthermore, the use of a defective variant of either LLO (ex. LLOS176W) or other pore-forming toxins should be strongly considered to determine whether or not this signaling pathway is truly a response to the pore-forming activities of this toxin instead of alternative mechanisms (ex. contaminating endotoxins that trigger an immune response).

The revised version of our manuscript now includes infection experiments with *L. monocytogenes* WT and Δhly (Figure S1A, B, E, F), demonstrating that tyrosine phosphorylation of NMHC2A during infection is triggered by LLO. We have also extended the analysis to additional PFTs, including purified PLY and PFO. To directly test the dependence on pore formation and calcium influx, we included a non–pore-forming PFO mutant and a calcium-free condition (Figure S2). Together, these data show that NMHC2A tyrosine phosphorylation specifically requires pore formation and the resulting calcium influx.

Given the results on Figures 5C and 5E, whether this pathway is a general stress response to the activation of innate immune pathways instead of pore-formation itself should be considered.

We thank the reviewer for raising this important alternative explanation. We have revised the Discussion to include the possibility that NMHC2A tyrosine phosphorylation may reflect a broader stress or innate immune response rather than pore formation alone (Page 14).

2. The results of the propidium iodide assay, which tests the essentiality of NMHC2A phosphorylation status to PM repair using flow cytometry, demonstrate a modest effect size, which are potentially not statistically significant using a statistical test that takes into account unequal sample variances. Furthermore, there is quite a significant number of cells permeable to propidium iodide at baseline (~12% despite having no exposure to LLO), which may suggest improper gating or poor growth conditions of the cells. Lastly, as mentioned above, the use of defective variants for LLO or other pore-forming toxins should be considered to evaluate whether or not this signaling response is truly a response to “LLO-induced damage.”

We appreciate the reviewer’s careful evaluation of the propidium iodide (PI) assay and the opportunity to clarify the raised issues.

First, we fully agree that the effects are moderate in magnitude. However, these experiments were deliberately performed using the highest concentration of LLO that still permits PM repair without inducing cell death. Under these conditions, we consistently observe relatively subtle quantitative differences. Based on our extensive experience with this assay ([Mesquita et al, 2017](https://www.embopress.org/doi/full/10.15252/embr.201642833); [Brito et al., 2019](https://www.mdpi.com/2072-6651/11/7/419); [Alves, Pereira et al., 2022](https://www.mdpi.com/2072-6651/15/1/4)) in the context of membrane damage and repair, even small shifts under tightly controlled, near-threshold injury conditions have repeatedly proven to be biologically meaningful. We now explicitly acknowledge the moderate effect in the revised manuscript and clarify the experimental rationale for using these conditions (Page 9).

Regarding the concern about baseline PI-positive cells in non-intoxicated samples, the observed ~10–12% PI positivity is primarily a consequence of cell processing required for flow cytometry, rather than reflecting impaired cell health, poor growth conditions, or improper gating. Specifically, to perform FACS analysis, cells must be trypsinized, washed, and resuspended prior to PI incubation. It is well documented that trypsin-mediated detachment by itself induces a measurable fraction of transiently permeabilized cells. A study directly comparing PI uptake following trypsinization versus mechanical detachment reports 10% PI-positive cells after trypsin treatment alone ([Yan et al., 2018](https://doi.org/10.21873/anticanres.13034)), which is in excellent agreement with our baseline values. In support to this baseline PI permeability being due to the cell processing is the fact that we obtained equivalent levels of PI positive cells in non-intoxicated untreated, Dasatinib-incubated, shCtr and ShSrc cells. In addition, the levels of PI positive cells consistently and significantly increased upon intoxication with LLO. Thus, all conclusions are based on direct quantitative comparisons between non-intoxicated and LLO-intoxicated conditions that undergo identical processing.

Finally, as mentioned above, we provide experiments with other PFTs thus extending the described pathway as a general response to pore formation.

Thus, additional experiments and a more transparent account of the flow cytometry gating ought to be provided to allow readers to better assess the accuracy of the claim. Suggested supplementary material can include:

a. Provide the gating strategies for Figure 3E and include a positive control (ex. digitonin-permeabilized cells) to allow readers to identify which cells are truly positive for propidium iodide.

We have included in the revised version (Figure S4) a representative gating strategy used for the analysis of the PI assays. In addition, we now include a positive control for maximal permeabilization (“100% lysis”) using 0.05% Triton X-100 to illustrate the expected profile of fully PI-positive cells. We do not include this control in every experimental replicate. Importantly, all gating was performed using unstained controls included in each run, which ensured consistent and experiment-specific discrimination of PI-positive versus PI-negative populations.

b. The use of transfected catalytically active or dead Src variants (as thoroughly provided in Fig. 2E) to assess positivity for propidium iodide (please see comment above to include control and gating strategies). Alternatively, use the NMHC2A variants in Brito, Pereira, Mesquita et al. 2023 (Ref. 24) to potentially complement information from Figure 3E.

We agree that using catalytically active or inactive Src variants, or NMHC2A phosphomutants, would in principle provide complementary mechanistic insight into the role of Src-dependent NMHC2A phosphorylation in PM repair. As shown in Figure 2E, we successfully expressed Src variants for biochemical signaling analyses. However, extending this strategy to the PI-based membrane repair assay proved technically challenging. Specifically, combining transient expression of Src or large GFP-tagged NMHC2A variants with subsequent LLO intoxication reproducibly resulted in excessive cell death, precluding reliable quantification of PI uptake. This limitation is consistent with our experience that the combined stress of transfection, high protein overexpression, and PFT challenge exceeds cell tolerance.

For this reason, we opted for genetic and pharmacological Src inhibition approaches (Src-deficient cells and dasatinib treatment), which allowed us to examine the requirement for Src activity under physiological expression levels and under conditions compatible with the PI assay. Through these approaches, cells impaired in Src expression or activity consistently showed increased PM permeability supporting a functional role for Src signaling in this process.

Regarding the use of NMHC2A mutants described in ([Brito et al, 2023](https://www.mdpi.com/2073-4409/12/14/1871)), we also attempted complementation strategies using GFP-tagged NMHC2A variants, as detailed in our response to point 3. However, similar to the Src mutant experiments, these cells did not tolerate the combination of NMHC2A depletion, re-expression of a large motor protein, and subsequent LLO challenge. We have now clarified this limitation explicitly in the revised manuscript.

c. Including a time-course of Figure 3E to document both positivity for propidium iodide over time and at increasing concentrations of LLO ( both WT and LLOS176W) and pair this with a measure of cell death (XTT, LDH, etc) assess whether or not components in this pathway truly contribute to PM repair and protection from pore forming toxins.

We thank the reviewer for this constructive suggestion. In the revised manuscript, we have now included an additional time-course assay (Figure S3B-D) that monitors PM permeability using Draq7, a DNA-impermeant dye functionally analogous to propidium iodide. Coupled with high-content microscopy (see Materials and Methods), this assay reveals a progressive increase in Draq7 positivity over time in cells exposed to sub-lytic concentrations of WT LLO (0.25–1 nM). Furthermore, these time-course experiments show that cells intoxicated with LLO (0.5 and 1 nM) exhibit significantly enhanced Draq7 uptake when treated with Dasatinib, compared to mock-treated controls. In contrast, non-intoxicated cells remain Draq7-negative regardless of Dasatinib treatment, confirming that dye incorporation strictly reflects LLO-induced membrane injury.

Importantly, we previously established ([Alves, Pereira et al., 2022](https://www.mdpi.com/2072-6651/15/1/4)) that the LLO concentrations used are sub-lytic and permit PM repair rather than inducing cell death. The new assay (Figure S3B-D) further confirms that the LLO concentrations used do not induce extensive cell death over the duration of the experiment, as the total number of nuclei (stained with Hoechst) in the end of the experiment remained similar across conditions, indicating that Draq7 positivity reflects transient membrane permeabilization rather than loss of cell viability. Of note, classical viability assays such as LDH release or metabolic readouts (e.g., XTT) are not ideal for quantifying cell viability in response to PFTs. LDH assays directly measure cytosolic enzyme leakage through membrane disruptions; thus, in the context of a PFT, increased LDH release may reflect pore formation rather than irreversible cell death. Likewise, metabolic assays can yield ambiguous results because PFTs can directly impair mitochondrial function ([Stavru et al., 2011](https://www.pnas.org/doi/full/10.1073/pnas.1100126108); [Stavru et al., 2013](https://doi.org/10.1073/pnas.1315784110)) without necessarily triggering cell death.

Regarding the inclusion of the LLOS176W mutant, we agree that a full comparison with this non-hemolytic mutant form could be informative. However, this mutant is not available in our laboratory, and obtaining and purifying it would exceed the time frame of the current review process. Since LLOS176W is well characterized as pore-deficient (Koster et al., 2014), we would expect its behavior in this assay to resemble that of non-intoxicated cells or cells treated with the lowest LLO dose, and thus it is unlikely to alter the conclusions drawn from the WT toxin.

Collectively, these results strengthen our conclusion that Src-dependent pathways contribute critically to cellular protection against PFT–induced PM damage.

3. Finally, the biochemistry with the phophomutants is robust in demonstrating the lack of impact on ATPase activity and actin binding, however it is not clear that these point mutations lead to defects in the cellular response to LLO as assumed. These constructs should be used to complement NMHC2A knockdown/knockout cells intoxicated with LLO to ensure that the model that these functions are not important for the response to LLO is valid.

We fully agree with the reviewer that complementation of NMHC2A-depleted cells with the phosphomutants would be the most direct approach to test the role of Tyr158 phosphorylation in the response to LLO and other PFTs. We attempted this strategy repeatedly using sequential NMHC2A knockdown followed by expression of GFP-tagged wild-type or mutant NMHC2A. However, despite multiple optimization attempts, the cells consistently exhibited extensive cell death during the intoxication step, precluding meaningful analysis. We believe that the combination of prolonged NMHC2A depletion, expression of a large (250 kDa) GFP-tagged motor protein, and subsequent PFT challenge exceeds the tolerance of these cells.

As an alternative and complementary strategy, we therefore used Src-deficient cells, in which Tyr158 phosphorylation is specifically impaired, to assess the contribution of this modification to the cellular response to LLO. This approach allows us to probe the functional relevance of Tyr158 phosphorylation in a physiological expression context without the confounding toxicity associated with forced re-expression.

**Part III – Minor Issues: Editorial and Data Presentation Modifications**
Please use this section for editorial suggestions as well as relatively minor modifications of existing data that would enhance clarity.

**Reviewer #1:**

1. Standardize nomenclature: the nematode specific toxin should be referred to as Cry5Ba (not Cry5B or Cry5b).

Corrected in the revised version.

2. Clarify whether the authors assessed possible effects of NMHC2A phosphorylation on interactions with regulatory light chains or actin-binding partners.

We thank the reviewer for raising this important point. In this study, we did not directly assess the effects of NMHC2A Y158 phosphorylation on its interactions with light chains. However, our data suggest this phosphorylation is unlikely to affect light chains binding. As shown in Figure S3A, the heavy meromyosin (HMM) fragments purified from the cells expressing WT and phosphorylation variants all contained comparable amounts of regulatory (RLC) and essential (ELC) light chains, suggesting that their association does not depend on the Y158 phosphorylation status. Furthermore, the viability of the *C. elegans* non-phosphorylatable mutant (this study) suggests that this mutation does not interfere with major protein–protein interactions required for NM2A function.

Regarding other potential binding partners, including actin-associated proteins, in the context of another study, we are currently trying to identify NMHC2A interactors under intoxication versus control conditions using a BioID approach. The data currently available are too preliminary to draw conclusions.

3. The specificity of the Dasa inhibitor must be more rigorously addressed; otherwise, conclusions about Src dependency remain open to question.

We thank the referee for raising this important point regarding the specificity of chemical inhibitors. We fully agree that specificity is a general limitation when using kinase inhibitors, and Dasatinib in particular is described to target both Src and Abl family kinases. Nonetheless, Dasatinib has been extensively used in research studies. To overcome the raised concern, we employed multiple complementary approaches beyond pharmacological inhibition:

**- Genetic knockdown** of Src expression, which resulted in phenotypes highly comparable to those observed with Dasatinib treatment.

**- Overexpression of mutant Src variants** (constitutively active and kinase-dead), which produced clear and opposite phenotypes in terms of NMHC2A phosphorylation.

**- Concordance of results**: the fact that all three independent strategies (pharmacological inhibition, genetic knockdown, and mutant overexpression) yielded consistent and robust effects strongly supports the conclusion that Src is required for NMHC2A phosphorylation.

We note that Reviewer #3 also highlighted the strength of this combinatorial strategy, emphasizing that the use of diverse approaches, provide strong support for the Src dependency of NMHC2A phosphorylation.

Furthermore, the use of multiple methods is a well-accepted strategy in the field to overcome the limitations of any single approach, particularly those associated with inhibitor specificity. In our view, the convergence of evidence from these different experimental angles provides sufficient rigor to validate the conclusions presented.

We have revised the manuscript text to explicitly acknowledge the limitations of inhibitor specificity and to highlight the complementary nature of the approaches we used (Page 8).

4. The shRNA strategy raises concerns about off-target effects. Appropriate rescue experiments or additional validation would be needed to rule these out.

We appreciate the reviewer’s concern regarding potential off-target effects associated with the shRNA strategy. It is true that such effects can occur; however, the shSrc-depleted cells used in this study have been employed in several previous experiments in our laboratory and are well characterized ([Almeida et al, 2015](https://pmc.ncbi.nlm.nih.gov/articles/PMC4375491/), [Brito et al, 2023](https://www.mdpi.com/2073-4409/12/14/1871)). Such cells consistently show a 60 to 70% reduction in the expression of Src (determined by western blot) and yielded reproducible phenotypes. In addition, as explained above, our conclusions are supported by independent experimental approaches, including the use of pharmacological inhibitors and the overexpression of mutant Src variants, both of which yielded results consistent with those obtained using shRNA-mediated depletion.

We agree that a rescue experiment would provide additional confirmation. However, in our system, such an experiment is technically very challenging, as it would require sequential transfection followed by intoxication of the Src-depleted cells, a combination that results in dramatic cell death and poor viability. Therefore, although a classical rescue experiment is not feasible in our system, the consistency between multiple experimental approaches serves as functional validation.

**Reviewer #2:**

• Fig. 1, please include loading control quantification or normalization to the loading control

We apologize for the misunderstanding. The Y-axis labels (“NMHC2A pTyr (a.u.)” and “Src activation (a.u.)) in the original version of the manuscript were misleading. The quantifications already included normalization to the signal of total NMHC2A or Src in the whole-cell lysate (WCL). Accordingly, we have corrected the Y-axis labels in the revised manuscript to “NMHC2A(IP pTyr/ WCL)” and “Src pTyr419/Src WCL”.

• Fig. 1C, please include a negative control such as a non-activated cell kinase

We thank the reviewer for this thoughtful suggestion. We understand the rationale for including an additional negative control, such as a non-activated cell kinase. However, in this context, we believe this control is not essential for interpreting the specific activation pattern of Src kinase. We are indeed investigating the behavior of several other kinases in response PFTs as part of ongoing parallel projects, but we prefer not to include those data here to maintain the focus and scope of the present manuscript. We hope the reviewer agrees that the current controls sufficiently support the conclusions drawn from Figure 1C.

• Fig. 1B and 1D, please use consistent statistical analyses, specifically ANOVA tests

We apologize for the confusion in the previous version of the manuscript. We incorrectly stated that an ANOVA was used for Figure 1D; in fact, a two-tailed unpaired Student’s *t*-test was performed. This has now been corrected (Legend of Figure 1).

In Figures 1B and 1D, we are comparing two groups (non-intoxicated versus LLO-intoxicated). To assess whether there is a statistically significant difference between two groups, a t-test is the standard approach. Alternatively, a one-way ANOVA can also be applied when comparing only two groups; in this case, it is mathematically equivalent to an independent t-test and yields the same p-value and conclusion. Therefore, either test is appropriate, and both lead to identical results in this context.

• Fig. 1E is slightly blurry and difficult to read

An improved, higher-resolution version of Figure 1E was included in the revised manuscript.

• Fig. 2, please include loading control quantification or normalization to the loading control

We apologize for the misunderstanding. As for Figure 1, the Y-axis labels (“NMHC2A pTyr (a.u.)) in the original version of the manuscript were misleading. The quantifications already included normalization to the signal of total NMHC2A in the whole-cell lysate (WCL). Accordingly, we have corrected the Y-axis labels in the revised manuscript to “NMHC2A (IP pTyr/ WCL”.

• Fig. 2B, 2D, 2F, and 2J please use consistent statistical analyses, specifically ANOVA tests

Statistical analysis of the data in Figure 2B and 2F was performed using a one-way ANOVA, while in Figure 2D and 2J a t-test was applied. The choice of test depended on the number of groups being compared. When only two groups are analyzed, a one-way ANOVA and a t-test are mathematically equivalent, yielding the same p-value and conclusion. The standard approach for comparing two groups is the t-test.

• Fig. 2G, the red and green words on the microscopy images is difficult to read

This was modified in the revised version.

• Fig. 2G, the arrowed regions in NI are not clear

Figure 2G has been modified in the revised version of our manuscript. Insets are now provided instead of arrows.

• Fig. 3A, please include the untreated control, shCtr (no LLO) control, and shSrc (no LLO) control, in addition to the quantification of the controls

In a previous study ([Brito et al., 2023)](https://www.mdpi.com/2073-4409/12/14/1871), we demonstrated that cells with reduced Src expression (shSrc) or activity (Dasa treatment) display increased actin stress fibers, as shown in Figure 2A and B of [Brito et al., 2023](https://www.mdpi.com/2073-4409/12/14/1871) (Figures provided below). However, no cortical accumulations of NMHC2A were detected under these conditions, which goes in agreement with our previously published data showing that the accumulations of NMHC2A at the cell cortex are dependent on pore-forming activity of LLO as well as other PFTs ([Mesquita et al., 2017](https://www.embopress.org/doi/full/10.15252/embr.201642833), [Brito et al., 2019](https://www.mdpi.com/2072-6651/11/7/419)).

As requested by the reviewer, confocal images of non-intoxicated shCtr, shSrc and Dasatinib-treated cells labelled for NMHC2A are now provided in the revised manuscript as Figure S3A.

Published figure Legend (Brito et al., 2023 ): Src kinase controls actomyosin cytoskeleton organization in HeLa cells. (A) Confocal microscopy images of control (mock and shCtr) and Src-impaired (Dasatinib-treated and shSrc) HeLa cells stained with phalloidin for f-actin (greyscale) and with DAPI (blue). Scale bar, 10 μm. (B) Percentage of cells with stress fibers quantified from images similar to (A). Values are the means ± SEM (n = 4); p-values were calculated using a two-tailed unpaired Student’s t-test, * p < 0.05, ** p < 0.01.

• Fig. 5C and 5E, please use ANOVA for statistical analyses

In Figures 5C and 5E, we compare two groups (wild-type worms versus Y163F mutant worms). As noted above, the standard test for this type of comparison is the t-test, which is mathematically equivalent to a one-way ANOVA with two groups and yields the same result.

• Fig. 5 results section, please include the rational for the heat stress assay

We thank the reviewer for this comment and the opportunity to clarify our rationale for including the heat stress assay in *C. elegans* mutants expressing the non-phosphorylatable NMY-2. Our study primarily focuses on the cellular response to membrane damage caused by pore-forming toxins. To determine whether the observed effects were specific to pore formation or reflected a more general stress response, we complemented the infection assays with a heat stress assay. Assessing heat stress sensitivity in *C. elegans* mutants is a widely used and well-established assay, making it a suitable complementary approach to evaluate general stress resilience. In addition, our previous work ([Mesquita et al., 2017](https://www.embopress.org/doi/full/10.15252/embr.201642833)) demonstrated that PFTs target the endoplasmic reticulum (ER) and can affect heat shock proteins, including the ER-resident chaperone Gp96. Given this connection, and considering that heat stress assays are a well-established and straightforward method in *C. elegans*, we found it an appropriate complementary approach to assess the robustness and specificity of the observed phenotypes. A sentence explaining this rationale has been included in the revised version of the manuscript (page 12).

• Introduction, 2nd paragraph, “internalization vacuoles” should be “the phagosome”

Corrected in the revised version.

• Pg. 7 typo “non-phophorylatable”

Corrected in the revised version.

• Pg. 12 typos “activationand” and “cells boundaries”

Corrected in the revised version.

• Discussion, please include additional information if available on other pore forming toxins and host cell pathway activation and whether this may be a general mechanism

In the revised version of the manuscript, we have included new data (Figure S2) on other PFTs from the same family as LLO (e.g., PFO and PLY). Accordingly, we have expanded the Discussion to address these results and to consider whether the observed effects may reflect a more general mechanism of host cell pathway activation by PFTs (page 13).

**Reviewer #3:**

1. The integrated density measurements in Figure J alone does not match the degree of enrichment of pY158F in Figures 2GH. Perhaps a ratio of the integrated density in the cortical vs an immediately sub-cortical region (noting the consistent presence of pY158F signal in the nuclear/perinuclear region) can better reflect the enrichment of the signal at the cortical region.

We agree with the reviewer that the quantification of pY158 enrichment does not fully reflect the strong signal observed in Figures 2G and H. This discrepancy likely arises because, in non-intoxicated cells, pY158 signal at focal adhesions also contributes strongly to the overall integrated density. We attempted several alternative quantifications but were unable to identify one that fully captured the visual enrichment seen in the images. Nevertheless, we found the reviewer’s suggestion to quantify an immediate sub-cortical region very helpful. We have now provided (new Figures 2IJ) quantifications for two regions: the cortical and the immediate sub-cortical regions. The pY158 signal is significantly higher in the cortical region of LLO-intoxicated cells, whereas it remains similar in the sub-cortical region of NI and LLO cells. These results indicate that NMHC2A phosphorylation occurs specifically at the cortical region of the cell, where the PM undergoes damage.

2. The authors’ efforts in plotting individual replicates via scatter plots for nearly all quantifications are noted and applauded. Plotting standard deviation instead of SEM in the line graph in Figure 5B would similarly better allow the reader to assess the data variance for this key experiment.

We thank the reviewer for the positive feedback. As recommended, in the revised version of the manuscript, the graph in Figure 5B now displays standard deviation (SD) instead of standard error of the mean (SEM). The figure legend has been updated accordingly.
