# Supplementary material for: Src-dependent tyrosine-phosphorylation of NM2A has a protective role against bacterial pore-forming toxins
Source: PLoS Pathog. 2026 Feb 23;22(2):e1013945. doi: 10.1371/journal.ppat.1013945 (PMC12959841; doi:10.1371/journal.ppat.1013945)
Supplement: Attachment — Submitted filename: Response to the reviewers-Revision 2- PloS Path.docx [file ppat.1013945.s014.docx]

**Point-by-Point response to the reviewers’ comments**

**Part I – Summary**

Please use this section to discuss strengths/weaknesses of study, novelty/significance, general execution and scholarship.

**Reviewer #2:** In “Src-dependent tyrosine-phosphorylation of NM2A has a protective role against bacterial pore-forming toxins”, Brito et al. the authors identify an unrecognized pore forming toxin (PFT) induced Src-dependent pathway critical for cytoskeletal reorganization and response to plasma membrane damage. The authors use Caenorhabditis elegans to show a possible conserved mechanism in response to pore forming induced stress and heat shock. This is an interesting story and the authors have addressed several of the major issues described during the first review. The use of other PFTs (PLY and PFO) in addition to LLO showing they trigger NMHC2A Ty158 phosphorylation in a pore forming and calcium influx dependent manner make this paper more significant suggesting this may be a general response to PFTs. The authors have also used a Listeria monocytogenes deltaLLO control to demonstrate that LLO is critical for Src and tyrosine phosphorylation of NMHC2A. Over-all the manuscript is much improved compared to the first submission.

**We thank Reviewer 2 for their thoughtful and constructive assessment, and we appreciate their positive evaluation of the revisions and recognition that the manuscript has been substantially improved and strengthened by the additional experiments.**

**Reviewer #3:** This revised manuscript has added significant improvements to address previous reviewer concerns. Notably the inclusion of data infecting with WT and ∆hly mutant Listeria and the addition of both other toxins and pore forming dead toxins significantly improve the argument that this signalling cascade is triggered by pore forming toxins and in the presence of bacterial infection. I still remain unconvinced that the authors have demonstrated membrane repair but rather have simply demonstrated differences in susceptibility to pore forming toxins which could be a result of increased pore formation and/or decreased pore repair. Language has been softened in the manuscript with regard to this point, however I think the authors could more clearly and explicitly state that their data is consistent with alterations in membrane repair but could also be indicative of increases in sensitivity of the cells. Finally, while I appreciate the authors attempting complementation with the biochemical mutants, the fact that these experiments were unsuccessful means that the authors need to be even more measured in their interpretation of the biochemical assays as they are simply unable to test whether these biochemical phenotypes are relevant in cells. In sum, while I believe the manuscript is significantly improved, these two aspects of the interpretation of the results are still somewhat overstated and could be softened to allow for the ambiguity that some of the data still contains.

**We thank Reviewer 3 for their careful and thoughtful evaluation of the revised manuscript and for acknowledging the substantial improvements made in response to the previous review. We appreciate the reviewer’s insightful comments regarding the interpretation of membrane repair and the limitations of the biochemical complementation experiments.**

**In response, we have further revised the manuscript to more clearly and explicitly temper our conclusions, emphasizing that our data are consistent with alterations in membrane repair but may also reflect differences in cellular sensitivity to pore-forming toxins (PAGE 8 of the revised manuscript).**

**We have also softened the interpretation of the biochemical assays to better reflect the experimental limitations and the remaining ambiguity in linking these biochemical phenotypes to cellular relevance (PAGE 10 of the revised manuscript).**

**Part III – Minor Issues: Editorial and Data Presentation Modifications**
Please use this section for editorial suggestions as well as relatively minor modifications of existing data that would enhance clarity.

**Reviewer #2:**

Please describe and provide a citation for Draq7, in the section where Draq7 is used.

**We thank the reviewer for this suggestion. A description of Draq7, along with the appropriate citation, has now been integrated into the revised manuscript (PAGE 8).**
